# Identification of recombination events in outbred species with next-generation sequencing data

**DOI:** 10.1186/s12864-018-4791-x

**Published:** 2018-05-25

**Authors:** Shentong Tao, Jiyan Wu, Dan Yao, Yuhua Chen, Wenguo Yang, Chunfa Tong

**Affiliations:** Co-Innovation Center for Sustainable Forestry in South China, College of Forestry, Nanjing Forestry University, No.159, Longpan Road, Xuanwu Qu, Nanjing, 210037 China

**Keywords:** Crossover, Gene conversion, Haplotype block, Next-generation sequencing, *Populus*

## Abstract

**Background:**

Meiotic recombination events include crossovers and non-crossovers or gene conversions. Although the rate of crossovers is often used for genetic mapping, the gene conversion events are not well studied especially in outbred species, which could produce distorted markers and thus affect the precision of genetic maps.

**Results:**

We proposed a strategy for identifying gene conversion events in *Populus* with the next-generation sequencing (NGS) data from the two parents and their progeny in an F_1_ hybrid population. The strategy first involved phasing the heterozygous SNPs of the parents to obtain the parental haplotype blocks by NGS analytical tools, permitting to identify the parental gene conversion events with progeny genotypes. By incorporating available genetic linkage maps, longer haplotype blocks each corresponding to a chromosome can be created, not only allowing to detect crossover events but also possibly to locate a crossover in a small region. Our analysis revealed that gene conversions are more abundant than crossovers in *Populus*, with a higher probability to generate distorted markers in the regions involved than in the other regions on genome. The analytical procedures were implemented with Perl scripts as a freely available package, *findGCO* at https://github.com/tongchf/findGCO.

**Conclusions:**

The novel strategy and the new developed Perl package permit to identify gene conversion events with the next-generation sequencing technology in a hybrid population of outbred species. The new method revealed that in a genetic mapping population some distorted genetic markers are possibly due to the gene conversion events.

**Electronic supplementary material:**

The online version of this article (10.1186/s12864-018-4791-x) contains supplementary material, which is available to authorized users.

## Background

Linkage mapping plays an important role in genetic analysis, especially in the context of quantitative trait locus (QTL) identification [1, 2], comparative genomics [3, 4], and genome scaffold sequence assembly [5, 6]. Since the publication of the first genetic map of *Drosophila melanogaster* [7], linkage maps have been constructed in many animal and plant species. Generally, a linkage map displays the linear order and genetic distance of molecular markers on chromosomes through analyzing the parental recombination events occurring during meiosis and passing on to the offspring. Thus, the precision and accuracy of linkage maps, which are crucial for applications, are affected by several factors, such as the mapping population size, the number and quality of markers, and the approach for ordering markers within a linkage group. In theory, the markers are required to be Mendelian factors, which segregate in a fixed ratio in a whole population. However, in practice, some markers were found not to follow Mendelian segregation, while the biological mechanics is not well explained up to date.

The *Populus* is a model system for forest trees. It has tremendous economic and ecological importance, and is widely distributed in North Hemisphere [8, 9]. A large number of linkage maps of different *Populus* species have been built in the past two decades using traditional molecular markers such as RAPD, RFLP, AFLP and SSR. In most of these studies, distorted markers that deviated from Mendelian segregation ratios were reported with frequencies varying from < 10% [10–14] to > 20% [15, 16]. Different strategies were taken for distorted markers when performing linkage analysis. Some studies excluded seriously distorted markers at the 1% significant level (i.e. *P* < 0.01) because these markers could bias linkage analysis and thereafter QTL identification [10, 13, 17, 18], while the others included all distorted markers as they were considered to be possibly associated with genes of interest [11, 14, 15]. The segregation distortion in *Populus* was generally believed to be related to many biological factors, such as genetic isolation, chromosome loss, genetic load, genome structural rearrangement, and linkage of markers to lethal genes [19, 20]. However, recent studies showed that gene conversion (GC) events during meiosis could be one of the main reasons that can skew segregation rates, but were typically ignored in genetic mapping studies [21].

It is well known that the result of meiotic recombination includes crossovers (COs) and non-crossovers (NCOs or GCs). COs reciprocally exchange DNA sequences between homologous chromosomes at a megabase scale, whereas GCs copy shorter sequences (less than a few kilobases) from one homologous chromosome to the other, altering allelic frequency [22–24]. Ky et al. [25] inferred that some distorted traditional markers of AFLP or RFLP in coffee may be due to GC events, but there were no direct evidences at genome-sequence scale. However, with the next-generation sequencing technologies, recent studies revealed that there are abundant GC events occurred during meiosis in *Arabidopsis*. Lu et al. [23] detected that the number of GCs was almost the same as the number of COs in *Arabidopsis*, like in yeast. Subsequently, however, Yang et al. [21] presented that more number of GC events were identified by sequencing 40 *Arabidopsis* plants and their parents with high coverage, rejecting the former estimate of equal numbers of CO and GC events.

In the present study, we investigated CO and GC events in *Populus* in order to understand the implications in generating distorted markers in a mapping population. We performed high-throughput whole genome sequencing of 10 progeny and their two parents in an F_1_ bybrid population of *Populus deltoides* and *P. simonii*. In a previous study [26], we have separately constructed the female *P. deltoides* and the male *P. simonii* linkage maps with thousands of single nucleotide polymorphisms (SNPs) generated by one of the next generation sequencing technologies. Here, a strategy was proposed to identify the CO and GC events in such a highly heterozygous tree species through the procedures: (1) phasing the parental haplotypes based on the reference genome of *P. trichocarpa* [9], (2) mapping the paired-end (PE) reads of each progeny to the reference sequences, (3) calling SNP genotypes and phasing for haplotypes for each individual, (4) identifying GCs by comparing the progeny haplotypes with the parents, (5) generating longer parental haplotypes with available linkage maps, and (6) finally forming progeny longer haplotypes with SNP genotypes and identifying COs in each progeny. The strategy was implemented with Perl scripts as a freely available package, *findGCO*, at the website of https://github.com/tongchf/findGCO. Consequently, 34.8 COs from the female parent and 27.3 from the male were found on average in progeny, while the numbers of GCs were 4055.6 and 3564.0, respectively, over 100 times the number of COs. Furthermore, we investigated the relationship between GCs and distorted markers with SNP data from 299 progeny in the same population, revealing that the distorted SNPs more frequently occurred in the regions of GCs than in the other regions. The results facilitated to recognize the role of GCs in forming distorted molecular markers and provided essential information when dealing with those markers in genetic mapping.

## Methods

### Plant materials and whole genome sequencing

An F_1_ full-sib family of *P. deltoides* × *P. simonii* was originally established as a mapping population. Approximately 500 progeny were planted in Xiashu Forest Farm of Nanjing Forest University, Jurong County, Jiangsu Province, China [26]. Ten progeny randomly chosen from the hybrid population as well as the two parents were considered as the materials for identifying recombination events in this study. Genomic DNA was extracted from fresh leaf tissue of each individual with the CTAB protocol [27]. Next, the qualified DNA was randomly sheared by sonication and Illumina adaptors with a unique multiplex identifier (MID) were added by ligation. A single library for the two parents with an insert size range of 300–500 bp was prepared and sequenced from both ends (paired-end, PE) with 101 bp read lengths in one lane of Illumina HiSeq 2000, while two libraries for the 10 progeny with the same insert size were constructed and sequenced (PE, 126 bp) in two lanes of Illumina HiSeq 2500. The whole-genome sequencing was performed at different times in Biomarker Technologies Co. Ltd., Beijing, China (BMK).

### Quality control and aligning of PE reads

The raw sequence data generated from the Illumina sequencers were filtered to obtain high-quality (HQ) reads with procedures as described in Mousavi et al. [28]. Briefly, we first discarded those PE reads that satisfy any one of the following conditions: (1) containing primer/adapter sequence, (2) having more than 10% uncalled bases (N), or (3) more than half of the bases in either of the reads having Phred quality score less than 5. The data generated from this step are called clean data. Secondly, the clean data were further filtered with NGS QC toolkit [29] to generate HQ reads such that the quality score is greater than or equal to 20 for ≥70% bases in either of PE reads.

In the process of haplotype phasing or SNP calling, PE reads of each sample including the two parents were required to align to the reference genome sequence of *P. trichocarpa* [9]. We used the command *mem* in the software of BWA [30] with default parameters to map the reads to the reference sequence, resulting in a SAM (sequence alignment/map) [31] file for each sample. To avoid to use those reads that are mapped to repeat regions in the reference, each SAM file was filtered such that each record in the file has an edit distance not more than 8% of the read length, with the best alignment score greater than or equal to 60 and the second-best alignment score less than the best alignment. After this step, the SAM file was converted to BAM format with SAMtools [31] for saving storage space and other subsequent analyses.

Removal of duplicate reads is a usual filtering step in processing NGS data for high quality. Duplicate reads are considered to be caused by multiple PCR products from the same DNA fragment, which may lead to false positive variant calls [32, 33]. We used the program MarkDupicaties in Picard package (http://broadinstitute.github.io/picard) to remove the duplicate reads contained in each BAM file generated above. The final processed BAM files were used for haplotype phasing analyses in the next sections.

### Parental haplotype construction

A haplotype is a linear set of bases from all SNPs in a given chromosome [34], and here we defined a haplotype block as a subset of bases in a haplotype. In diploid organisms, the recombination events at meiosis can be discovered by comparing the haplotypes of an individual and its parents. We used the command *phase* in SAMtools [31] with a minimum base quality score of 20 in heterozygote to obtain the information of the parental haplotypes using the corresponding BAM files created above. The records of this step were filtered to generate haplotype blocks for each parent. Each block must contain at least 5 SNPs with a coverage depth of at least 5 reads at each site and at least 3 reads for each allele. Furthermore, the genotype of each SNP in a parental block was required to be heterozygous in the current parent and homozygous in the other, and each genotype quality must have a Phred-scaled score of at least 60. In the following steps, for simplicity, the allele of homozygote for all SNPs in these haplotype blocks is denoted by ‘a’ and the other allele of heterozygote by ‘b’.

**Fig. 1:**
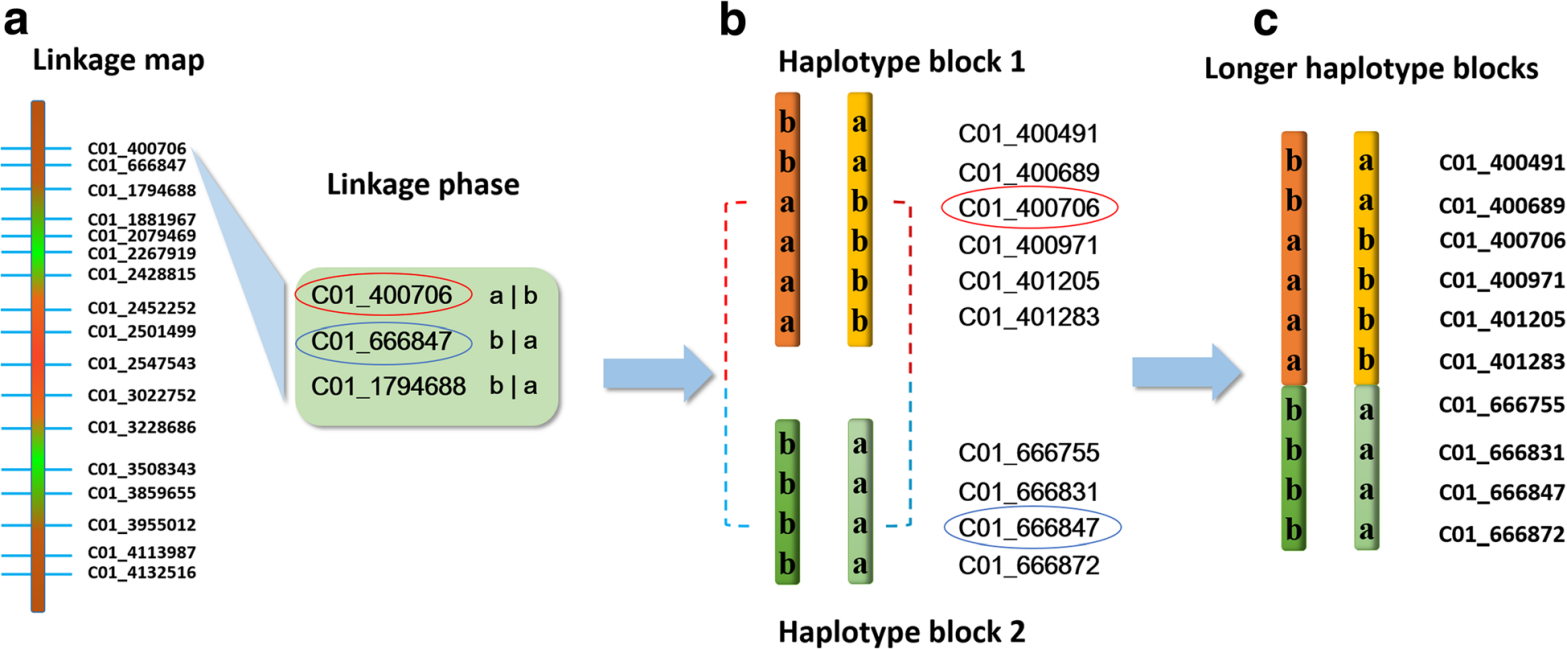
Merger of two haplotype blocks with linkage information. Two different alleles are denoted by ‘a’ and ‘b’ for all SNPs in the haplotype blocks. **a** The two SNPs, namely C01_400706 and C01_666847 with a repulsion linkage phase, are found in (**b**) haplotype blocks 1 and 2. **c** According to the linkage phase, the two blocks can be merged into a longer one (dark brown), with another one on the homologous chromosome

In order to obtain longer haplotype blocks, we used the linkage phase information of SNPs on the two parent-specific linkage maps constructed in the previous study [26] to merge two adjacent haplotype blocks on genome. The merging procedures can be described as in Fig. 1. When two SNPs on a linkage group with a known linkage phase (Fig. 1a) are found in two different haplotype blocks (Fig. 1b), the two haplotype blocks can be merged into a longer one, with another one on the homologous chromosome (Fig. 1c). Finally, each linkage group of two parental maps corresponded to a long haplotype block.

### Identification of recombination events

We called genotypes for each progeny at all SNPs contained in the haplotype blocks of the two parents. First, the command *mpileup* in SAMtools was used to generate BCF files, with each BAM file as input and the parameter of minimum base quality taking the value of 20. Second, a VCF file was produced with each BCF file using the command *call* of BCFtools (v1.1), which is accompanied with SAMtools, just skipping indels (insertions/deletions). Finally, each VCF file was filtered such that an SNP genotype has a sequence depth of at least 15 reads with the genotype mapping quality of greater than 60 and each allele coverage of at least 5 reads.

**Fig. 2:**
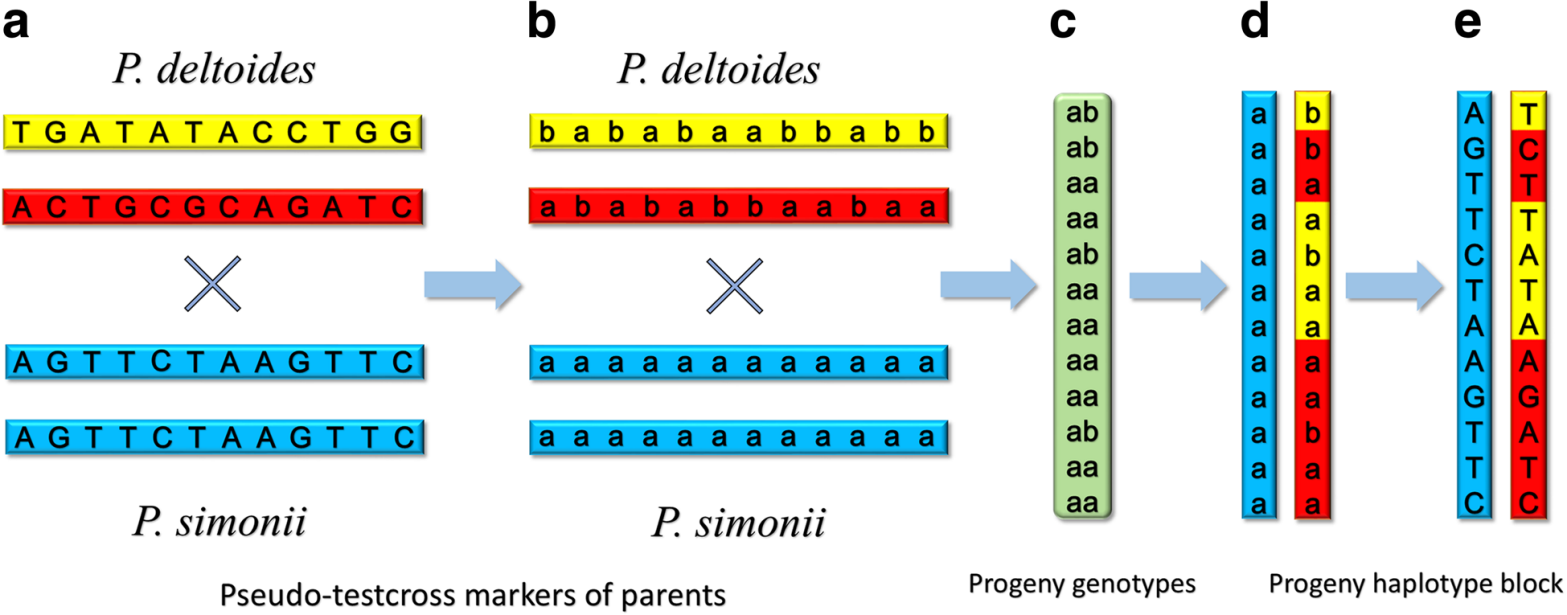
Procedures for identifying recombination events with a haplotype block inherited from one parent. **a** The haplotype blocks at the same SNP sites for two parents are shown. At these sites, the genotypes are all heterozygous in the female *P. deltoides*, but homozygous in the male *P. simonii*. **b** The homozygous allele is denoted by ‘a’ and the other allele in a heterozygote by ‘b’ at each SNP. **c** One progeny is genotyped with notations of ‘aa’ and ‘ab’ at those SNPs. **d** The haplotype blocks of this progeny can be discriminated, one (blue) from the male parent and the other (yellow/red) from the female. The haplotype block from the female carries recombinant information, in which the first red fragment (< 2 kb) from the top is considered to be a product of gene conversion and the junction between the second yellow and red fragments (> 10 kb) a crossover. **e** The alleles on the haplotype blocks of the progeny are labelled with base notations as they were

For each parental haplotype block, including the longer ones constructed with linkage information, we chose those SNPs at which the genotypes of the other parent are all homozygous (Fig. 2a and b). Those SNPs have the characteristic of pseudo-testcross markers [35], which can be used to identify recombination events in the progeny haplotypes as performed by Yang et al. [21] (Fig. 2c, d and e). At those pseudo-testcross SNPs, if the genotypes of a progeny are denoted by ‘aa’ or ‘ab’, the two haplotype blocks can be inferred, one of which is inherited from one parent with alleles of ‘a’s and the other from the other parent with alleles of ‘a’s and ‘b’s (Fig. 2c and d). Comparing the haplotype block containing ‘a’s and ‘b’s with the two in the heterozygous parent (Fig. 2b), the recombination events can be identified at meiosis in this parent. If a DNA fragment is less than 2 kb but greater than 20 bases and replaces a homologous sequence, a GC is thought to occur during meiosis [24, 36]; however, when two DNA fragments in lengths of more than 10 kb come from different homologous chromosomes and join together in a progeny haplotype block, a crossover is considered to exist at the junction [21, 22] (Fig. 2d).

## Results

### Reads quality control, mapping and duplicates removing

We sequenced the whole genomes using the platforms of Illumina HiSeq 2000 for the two parents, *P. deltoides* and *P. simonii*, and Ilumina HiSeq 2500 for the 10 progeny in BMK at different times. With the standard quality control (QC) pipeline at BMK, a total of 62.12 Gb clean data with a read length of 101 bp were obtained from the two parents and an average of 13.77 Gb with a read length of 126 bp from the progeny (Table 1). These clean data are available under accession numbers SRP071167 for the parents and SRP125267 and SRP125268 for the progeny at the NCBI Sequence Read Archive database (http://www.ncbi.nlm.nih.gov/Traces/sra). After a serious filtering process with NGS QC toolkit, the HQ reads data were obtained with a reducing range from 6 to 20%, in which over 96% bases have a Phred quality score of at least 20 for each individual (Table 1).

**Table 1:** Summary of the sequencing data in aspects of quality, mapping results and duplicate reads for the two parents and their 10 progeny

Sample ID	Clean PE reads (M)	Clean Bases (Gb)	HQ PE reads (M)	HQ bases (Gb)	Mapped reads (%)	Uniquely mapped reads^a^ (%)	Duplicate reads^b^ (%)	Remained reads (M)
P1^c^	144.06	29.10	117.67	23.77	96.15	57.40	12.69	48.19
P2^d^	163.46	33.02	131.28	26.52	95.67	59.27	33.99	37.43
B35–2	54.45	13.72	48.41	12.20	97.33	55.55	7.03	50.27
C25–3	51.93	13.09	46.09	11.60	97.78	55.91	5.26	51.80
C3–2	48.75	12.29	43.48	10.96	97.84	55.55	5.54	51.34
C32–2	52.76	13.29	46.86	11.81	97.85	55.84	3.92	52.50
C5–3	56.36	14.20	50.19	12.65	97.69	55.66	5.26	51.51
3–12	55.94	14.09	52.09	13.13	99.58	57.27	10.19	51.22
3–14	53.10	13.37	47.56	11.98	99.47	56.05	17.32	46.10
3–15	55.79	14.05	52.02	13.11	98.52	60.15	11.78	52.28
3–16	58.35	14.70	54.67	13.78	98.84	57.08	20.49	44.86
3–18	58.96	14.85	55.15	13.90	98.40	57.01	22.05	43.73

^a^The percentage of almost uniquely mapped reads in all mapped reads for a sample

^b^The percentage of duplicate reads in uniquely mapped reads for a sample

^c^P1, the female parent *P. deltoides*

^d^P2, the male parent *P. simonii*

We mapped the HQ reads of each individual to the reference genome of *P. trichocarpa*. As a result, 96.15 and 95.67% of the HQ reads from the female and male parents were aligned to the reference, respectively, and the mean percentage for the progeny was 98.33 with a standard deviation of 0.77. We filtered these mapped reads such that the edit distance is at most 8% of the single read length with the best alignment score of at least 60 higher than the second-best alignment score. The remaining reads were considered to be almost uniquely mapped to the reference genome [26], occupying 55.55–60.15% of the mapped reads of each sample. After that, we further removed the duplicate reads from these almost uniquely mapped reads, leading to 3.92–33.99% of the reads discarded for each individual. Consequently, 37.43–52.50% of the HQ reads of each individual, which were expectedly mapped to unrepeated regions with a maximum edit distance of 8 for the parents and 10 for the progeny, were remained for inferring haplotype blocks and identifying recombination events in the next section (Table 1).

### Construction of parental haplotype blocks

With the remained reads generated above, we used the command *phase* in SAMtools to call haplotype blocks for each parent. After performing the filtering steps as described in Materials and Methods, we obtained 54,753 haplotype blocks containing 647,971 HQ SNPs in the female parent of *P. deltoides*, while in the male *P. simonii* the number of haplotype blocks was 35,458 with a total number of 427,863 HQ SNPs (in Additional file 1: Table S1). These female and male blocks have the average spanned lengths of 842 and 806 bp with the longest lengths of 26,133 and 20,230 bp, totally covering 10.62 and 6.58% of the reference genome, respectively. By incorporating the genetic linkage maps, 19 longer haplotype blocks were constructed for each parent. The female longer blocks contained 9942 SNPs, of which 1205 SNPs are included in the linkage map, whereas the male longer ones contained 8149 SNPs with 700 from the male linkage map (in Additional file 1: Table S2). On each of the female haplotype blocks, including the longer ones, all the SNP segregation types are *ab*×*aa*, i.e., the female parent genotype is a heterozygote *ab*, but the male is a homozygote *aa* at each SNP site. On the contrary, the segregation types of all the SNPs on each male haplotype block are *aa*×*ab*.

### Identification of recombination events

**Fig. 3:**
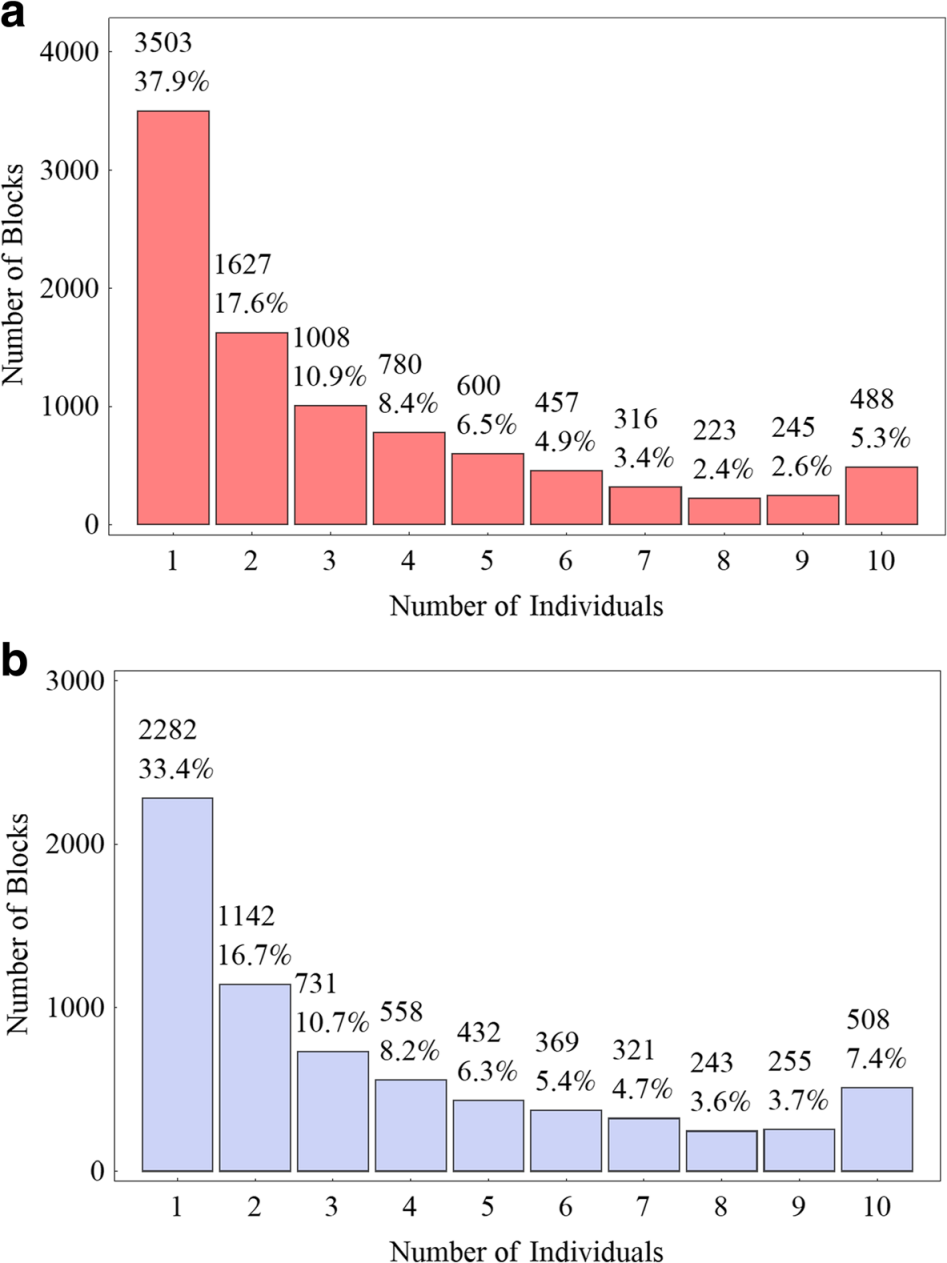
Bar charts of the number of haplotype blocks against the number of individuals in which one or more GC events were detected in the same haplotype block for the female (**a**) and male (**b**) parents

We compared the parental haplotype blocks with the blocks of each progeny to identify recombination events. As a result, 9247 (16.9%) of the maternal haplotype blocks and 6841 (19.3%) of the paternal were found to have recombination events detected in at least one progeny. We categorized these haplotype blocks according to the number of individuals in which one or more recombination events were detected in the same haplotype block. Figure 3 presented the bar charts of these categories for both parents. It can be seen that over 60% of these blocks have recombination events detected in at least two individuals, with over 5% (488/508) having recombination events identified in all the 10 progeny.

Table 2 presented the distribution of the number of recombination events identified in the progeny over fragment length. It is easily found that the over 95% of the recombination events belonged to GC (20 bp – 2 kb), while less than 2% could result from crossover events (> 2 kb). Table 3 and in Additional file 1: Table S3 presented the numbers of gene conversion events distributed on the reference genome sequences, which were identified in each of the 10 progeny and occurred during meiosis in the female and male parents, respectively. On average, we found 4055.6 maternal GCs with an average length of 231.4 bp and 3564.0 paternal GCs with almost the same average length (231.5 bp) in the progeny (in Additional files 2 and 3: Excel Sheets S1 and S3). Furthermore, we discovered that there existed strong correlations among the parental GC numbers and the lengths of the reference chromosomes of *P. trichocarpa*, with coefficients of 0.9606 between the female and the male, 0.9377 between the female and the reference and 0.9072 between the male and the reference.

**Table 2:** Distribution of the average number of recombination events occurred in the female (male) meiosis and identified in progeny over fragment length

Fragment length	2−19 bp	20−200 bp	200 bp−1 kb	1−2 kb	2−10 kb	≥10 kb
Chr01	26.4 (19.4)	373.9 (350.5)	192.8 (192.8)	14.2 (12.1)	3.7 (3.4)	0.0 (0.0)
Chr02	4.3 (9.4)	135.1 (109.6)	62.9 (53.7)	5.7 (4.9)	3.2 (1.6)	0.0 (0.0)
Chr03	10 .0 (10.0)	131.3 (137.9)	70.4 (66.3)	5.5 (5.9)	0.8 (0.3)	0.0 (0.0)
Chr04	8.9 (9.2)	127.3 (129)	68.5 (62.3)	5.5 (4.4)	1.5 (1.2)	0.0 (0.0)
Chr05	8.8 (8.4)	145 (108.2)	83.0 (54.8)	6.3 (5.0)	0.6 (0.5)	0.0 (0.0)
Chr06	6.9 (7.0)	131.8 (115.6)	69.2 (67.5)	6.3 (3.9)	3.0 (1.3)	0.0 (0.0)
Chr07	8.8 (4.8)	110.0 (80.5)	52.3 (34.6)	2.9 (2)	1.2 (0.6)	0.0 (0.0)
Chr08	8.8 (3.9)	92.9 (86.3)	62.1 (47.5)	7.7 (5.6)	0.8 (1.5)	0.0 (0.0)
Chr09	4.8 (4)	56.5 (57.7)	30.0 (31)	1.5 (1.9)	0.6 (0.1)	0.0 (0.0)
Chr10	5.1 (5.2)	148.0 (87)	80.6 (48.7)	5.5 (3.2)	1.5 (2.7)	0.0 (0.1)
Chr11	10.8 (4.9)	119.1 (110)	61.1 (49.8)	5.8 (2.9)	0.6 (0.9)	0.0 (0.0)
Chr12	8.0 (2.1)	103.6 (63.2)	43.6 (39.2)	4.4 (2.3)	1.6 (0.4)	0.0 (0.0)
Chr13	5.5 (5.9)	99.8 (93.8)	59.0 (47.4)	6.2 (5.2)	2.0 (1.3)	0.0 (0.0)
Chr14	5.3 (9.3)	129.7 (103.6)	55.0 (84.9)	4.1 (9.7)	2.5 (1.1)	0.0 (0.0)
Chr15	6.8 (6.4)	97.9 (86.3)	46.0 (47.3)	3.8 (2.3)	1.1 (1.7)	0.0 (0.0)
Chr16	26.4 (19.4)	373.9 (350.5)	192.8 (192.8)	14.2 (12.1)	3.7 (3.4)	0.0 (0.0)
Chr17	4.3 (9.4)	135.1 (109.6)	62.9 (53.7)	5.7 (4.9)	3.2 (1.6)	0.0 (0.0)
Chr18	10.0 (10.0)	131.3 (137.9)	70.4 (66.3)	5.5 (5.9)	0.8 (0.3)	0.0 (0.0)
Chr19	8.9 (9.2)	127.3 (129)	68.5 (62.3)	5.5 (4.4)	1.5 (1.2)	0.0 (0.0)
Scaff.	8.8 (8.4)	145.0 (108.2)	83.0 (54.8)	6.3 (5)	0.6 (0.5)	0.0 (0.0)
Total	6.9 (7.0)	131.8 (115.6)	69.2 (67.5)	6.3 (3.9)	3.0 (1.3)	0.0 (0.1)

**Table 3:** Distribution of the number of gene conversion events detected in each of the 10 progeny and inherited from the female parent based on the reference genome sequences

Ref.	Progeny ID										Aver.
	B35–2	C25–3	C3–2	C32–2	C5–3	3_12	3_14	3_15	3_16	3_18	
Chr01	561	547	553	544	564	556	560	763	555	606	580.9
Chr02	199	198	201	199	195	207	182	267	204	185	203.7
Chr03	196	215	216	194	200	191	201	259	181	219	207.2
Chr04	227	180	184	186	194	184	188	281	199	190	201.3
Chr05	241	247	210	228	216	231	230	277	222	241	234.3
Chr06	208	202	178	209	177	196	196	274	213	220	207.3
Chr07	169	148	156	170	169	164	152	226	153	145	165.2
Chr08	156	158	155	166	150	148	178	200	150	166	162.7
Chr09	89	84	93	93	76	94	70	105	103	73	88.0
Chr10	243	222	225	225	207	203	235	277	257	247	234.1
Chr11	174	161	198	172	172	176	187	227	202	191	186
Chr12	128	155	171	139	151	132	134	197	155	154	151.6
Chr13	156	161	184	157	161	148	138	215	171	159	165.0
Chr14	176	179	167	165	204	192	190	244	176	195	188.8
Chr15	144	136	144	134	130	138	149	200	160	142	147.7
Chr16	126	143	146	138	140	150	156	215	147	136	149.7
Chr17	162	169	170	166	159	152	173	213	161	178	170.3
Chr18	186	147	141	148	146	149	177	238	167	154	165.3
Chr19	241	219	207	227	195	231	229	205	247	234	223.5
Scaff.	204	241	212	221	219	246	198	226	237	226	223.0
Total	3986	3912	3911	3881	3825	3888	3923	5109	4060	4061	4055.6

**Fig. 4:**
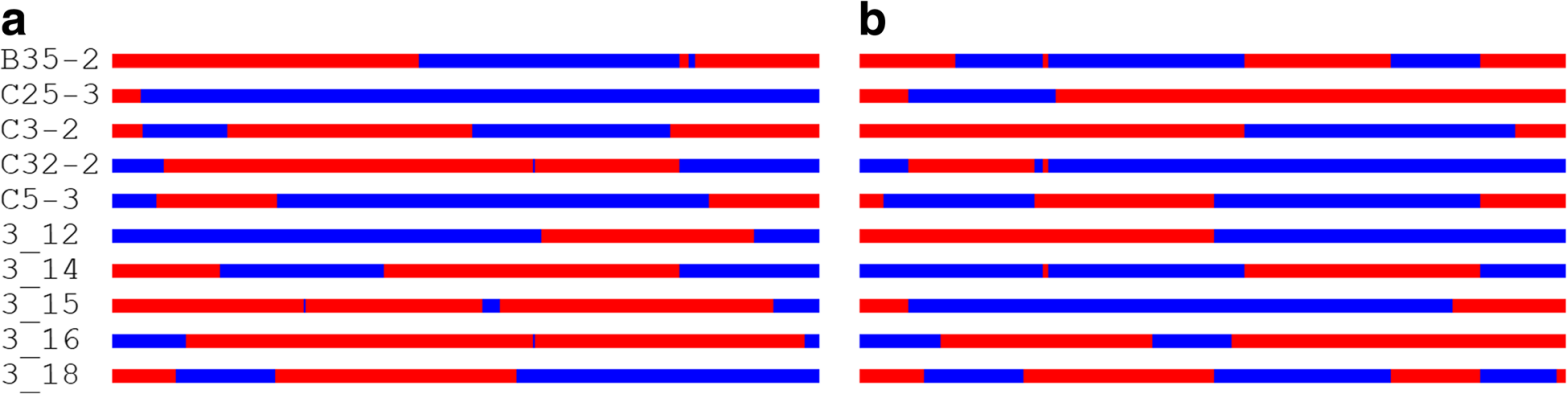
CO patterns identified in each progeny on chromosome 1 for the female (**a**) and male (**b**) parents. The red and blue bars represent two homologous chromosomes and the junction between the two colors is detected as a CO location

With the long haplotype blocks constructed by the parental linkage maps, CO events were identified from the SNP genotypes of each progeny at the sites of those long haplotypes. The distribution of CO events on chromosome 1 per parental meiosis was shown in Fig. 4 for the two parents. For other chromosomes, the CO patterns were presented in detail in Additional file 1: Figure S1 and S2. The spans formed by the two parental COs were given for all chromosomes of each progeny in Excel Sheets CD-B35–2 to CD-3-18 in Additional file 4 and Excel Sheets CS-B35–2 to CS-3-18 in Additional file 5. It can be calculated that an average number of CO events was 34.8 and 27.3 per meiosis in the female and male parents, respectively. In contrast, these numbers of COs are less than 1% of the GC numbers per meiosis.

### Implication of gene conversion for distorted SNPs

In order to investigate into the implication of GCs for distorted SNP markers, we analyzed two SNP datasets of different segregation types of *ab*×*aa* and *aa*×*ab*, which were generated from 299 individuals in the same F_1_ hybrid population of *P. deltoides* × *P. simonii* in the previous study of ours [26]. We filtered those SNPs such that at least 100 individuals have been genotyped, and the filtered SNPs were then classified into two categories, within or outside the GC regions that were identified in the 10 progeny in this study. Consequently, 367 SNPs in the *ab*×*aa* dataset and 380 in the *aa*×*ab* dataset were found in the GC regions, and the ratios of seriously distorted SNPs (*P <* 0.01) in these GC regions were 69.75 and 74.74%, respectively (Table 4). If the GC regions were limited to those that each was identified in at least 5 different individuals, the ratios of the distorted SNPs increased up to 84.84 and 76.38%, respectively. On the other hand, 17,640 of the filtered SNPs in the *ab*×*aa* dataset were found outside the GC regions with a distorted ratio of 31.35%, while in the *aa*×*ab* dataset there were 10,915 filtered SNPs outside the GC regions with a distorted ratio of 35.10%. Overall, the ratio of distorted SNPs within the GC regions was about two times greater than that outside the GC regions.

**Table 4:** The ratio of distorted SNPs within or outside GC regions detected in the current study for the SNP datasets of two different segregation types generated in the previous study (Tong, et al., 2016)

Segregation type	Region	SNP	Distorted^c^	Percent (%)
*ab*×*aa*	Non-GC	17,640	5530	31.35
	GC^a^	367	256	69.75
	GC5^b^	99	84	84.84
*aa*×*ab*	Non-GC	10,915	3831	35.10
	GC	380	284	74.74
	GC5	127	97	76.38

^a^GC, the region within GCs identified in at least one individual

^b^GC5, the region covered by GCs identified in at least 5 different individuals

^c^The seriously distorted SNP has a *p*-value of less than 0.01 with the Chi-squared test

## Discussion

We proposed a strategy for identifying recombination events during the meioses of the two parents in an F_1_ hybrid population of *P. deltoides* and *P. simonii* with the NGS technology. Unlike in the previous studies in *Arabidopsis* [21, 23], where the SNP phases of parents were known due to the inbred lines available, this strategy first needed to phase the heterozygous SNPs of parents to form the parental haplotype blocks by the NGS analytical tools such as BWA and SAMtools [30, 31]. Next, the specific parental haplotype blocks, in which all the SNP genotypes are heterozygous for one parent and homozygous for the other, were chosen and compared with the progeny haplotype blocks to identify the recombination events. Therefore, the method presented here permits us to explore especially the GC events in outbred species, which could affect segregation ratio of molecular markers involved and totally ignored in most previous genetic linkage analyses. Most importantly, we developed a Perl package to implement the complicated computing procedures, making it easy to identify recombination events using NGS data with just one keystroke.

The key step to implement the strategy is to phase the parental haplotype blocks of the outbred parents with NGS data and analytical tools. Here, as a primary work to investigate into the GC events in an outbred species, we directly applied the phase module in SAMtools to obtain the parental haplotypes, because this software contains many powerful functions and meanwhile, we also used it in this study for dealing with the reads mapping files in BAM format and calling SNPs, etc. Certainly, there are many other package tools available for haplotype phasing, such as fastPHASE [37], Beagle [38], SHAPEIT [39] and Eagle [40], which were mostly developed for medical and population genetics. A few of these phasing packages can handle NGS data with a reference sequence and could be utilized to improve accuracy of the parental phasing results in the current study. However, the phasing step for parental haplotypes would be skipped if we had an hybrid population produced by crossing an F_1_ individual with one of its parents (Backcross) or another F_1_ individual (F_2_). We noticed that in the previous study of ours [26], about 80% SNPs belong to the segregation pattern of *aa*×*bb* in the same F_1_ hybrid population of *P. deltoides* and *P. simonii*. These SNPs will segregate in the backcross or F_2_ hybrid population, having the same characteristics as in the usual experimental populations generated from inbred lines. Thus, if these SNPs are applied to the individuals from the backcross or F_2_ population, it will be very easy to identify recombination events as conducted in *Arabidopsis* [21].

Although the numerous phased parental haplotypes were not so long that the average length was ~ 800 bp and the longest one less than 30 kb, they indeed permitted us to explore the rich world of GC events for the first time in forest tree species. As performed by Yang et al. [21], we considered that the GC tracts are of 20 bp to 2 kb long and each must contains 2 SNPs, ignoring very short (< 20 bp) and long (> 2 kb) tracts, which amount to be less than 5% of the total (< 10 kb, Table 2). With the filtering procedures for mapped reads (see Methods), the identified GC events largely could be considered to be located in non-repeat regions on genome. Our analysis revealed that at least 99% of recombination events could be regarded as GCs, basically consistent with the findings in *Arabidopsis* [21]. Moreover, the GCs seemed not to randomly occur on the genome because the GC regions shared by all the 10 individuals have a higher frequency (> 5%) than those shared by 7, 8, or 9 individuals (< 5%, Fig. 4). This suggested that some genomic regions may have a preference for GCs, more possibly leading to the internal markers distortedly segregating in a mapping population. We validated it by analyzing the real SNP datasets from the previous study of ours (see Results).

By incorporating the linkage maps of the two parents, the longer haplotypes for the two parents and their progeny can be obtained that each corresponded to a chromosome, permitting to determine the positions of CO events with higher resolutions. Although a CO event could be identified at a marker interval of a linkage map just through the genotypes of an individual at those markers involved [41], the longer haplotypes constructed here could shorten the CO region if the flanking markers are within parental haplotype blocks. Occasionally, we could find a crossover within a parental haplotype block. We presented such cases for the two parental COs in Additional files 5 and 6: Excel Sheets S6 and S8 and, each of which was identified within a haplotype block and in a marker interval of 13–5329 bp in length. These high-resolution findings for COs provided important clues for further validating for some special proposes by sequencing target regions with the traditional Sanger sequencing or the single-molecule real-time (SMRT) sequencing recently developed by PacBio [42].

## Conclusions

The proposed novel strategy and the corresponding Perl package developed here permit to identify gene conversion events with the next-generation sequencing data in a hybrid population of outbred species. Our analysis revealed that in a genetic mapping population some distorted genetic markers are possibly due to the gene conversion events. More careful attention should be paid to the distorted markers when performing genetic linkage analysis.

## Additional files

Additional file 1: **Table S1.** Summary of parental blocks from the intermediate files of ‘parent1.abxaa.5snps.blocks’, ‘parent2.aaxab.5snps.blocks’, ‘parent1.long.haplotype’ and ‘parent2.long.haplotype’, created by *findGCO*. **Table S2.** The number of SNPs contained in the long parental haplotypes from the intermediate files of ‘parent1.long. Haplotype’ and ‘parent2.long.haplotype’ created by *findGCO*. **Table S3.** Distribution of the number of gene conversion events detected in each of the 10 progeny and inherited from the male parent based on the reference genome sequences. **Figure S1.** CO patterns identified in each progeny on all chromosomes in the female parent *P. deltoides*. **Figure S2.** CO patterns identified in each progeny on all chromosomes in the male parent *P. simonii*. (DOCX 277 kb)

Additional file 2: Excel Sheets RD-B35–2, RD-C25–3, RD-C3–2, RD-C32–2, RD-C5–3, RD-3-12, RD-3-14, RD-3-15, RD-3-16 and RD-3-18 Distribution of the number of recombination events over fragment length, which occurred in the meiosis of the female *P. deltoides* and were identified in each of the 10 progeny. Excel Sheet S1 Distribution of the average number of recombination events over fragment length, which occurred in the meiosis of the female *P. deltoides* and were identified in the 10 progeny. Excel Sheet S2 Summary of the number and the total length of haplotype blocks in which the maternal recombination events were identified in each progeny. (XLSX 29 kb)

Additional file 3: Excel Sheets RS-B35–2, RS-C25–3, RS-C3–2, RS-C32–2, RS-C5–3, RS-3-12, RS-3-14, RS-3-15, RS-3-16 and RS-3-18 Distribution of the number of recombination events over fragment length, which occurred in the meiosis of the male *P. simonii* and were identified in each of the 10 progeny. Excel Sheet S3 Distribution of the average number of recombination events over fragment length, which occurred in the meiosis of the male *P. simonii* and were identified in the 10 progeny. Excel Sheet S4 Summary of the number and the total length of haplotype blocks in which the paternal recombination events were identified in each progeny. (XLSX 29 kb)

Additional file 4: Excel Sheets CD-B35–2, CD-C25–3, CD-C3–2, CD-C32–2, CD-C5–3, CD-3-12, CD-3-14, CD-3-15, CD-3-16 and CD-3-18 Crossover tracts on each chromosome of the maternal *P. deltoides* that were identified in each progeny. Excel Sheet S5 Summary of the crossover numbers on the female chromosomes that were identified in each progeny. Excel Sheet S6 Summary of the crossover events on the female chromosomes that were identified within a short haplotype block region. (XLSX 72 kb)

Additional file 5: Excel Sheets CS-B35–2, CS-C25–3, CS-C3–2, CS-C32–2, CS-C5–3, CS-3-12, CS-3-14, CS-3-15, CS-3-16 and CS-3-18 Crossover tracts on each chromosome of the maternal *P. simonii* that were identified in each progeny. Excel Sheet S7 Summary of the crossover numbers on the male chromosomes that were identified in each progeny. Excel Sheet S8 Summary of the crossover events on the male chromosomes that were identified within a short haplotype block region. (XLSX 58 kb)

## Abbreviations

BMK: Biomarker Technologies Co. Ltd., Beijing, China
CO: Crossover
GC: Gene conversion
HQ: High-quality
NGS: Next-generation DNA sequencing
PE: Paired-end
QC: Quality control
QTL: Quantitative trait locus
SAM: Sequence alignment/map
SNP: Single nucleotide polymorphism

## References

[1] Lander ES, Botstein D (1989). Mapping Mendelian factors underlying quantitative traits using RFLP linkage maps. Genetics.

[2] Zeng Z-B (1994). Precision mapping of quantitative trait loci. Genetics.

[3] Krutovsky KV, Troggio M, Brown GR, Jermstad KD, Neale DB (2004). Comparative mapping in the *Pinaceae*. Genetics.

[4] Kakioka R, Kokita T, Kumada H, Watanabe K, Okuda N (2013). A RAD-based linkage map and comparative genomics in the gudgeons (genus *Gnathopogon, Cyprinidae*). BMC Genomics.

[5] Bartholome J, Mandrou E, Mabiala A, Jenkins J, Nabihoudine I, Klopp C (2015). High-resolution genetic maps of *Eucalyptus* improve *Eucalyptus grandis* genome assembly. New Phytol.

[6] Fierst JL. Using linkage maps to correct and scaffold de novo genome assemblies: methods, challenges, and computational tools. Front Genet. 2015; 10.3389/fgene.2015.002%2020. 10.3389/fgene.2015.00220 PMC4473057 26150829

[7] Sturtevant AH (1913). The linear arrangement of six sex-linked factors in *Drosophila*, as shown by their mode of association. J Exp Zool.

[8] Geraldes A, Pang J, Thiessen N, Cezard T, Moore R, Zhao Y (2011). SNP discovery in black cottonwood (*Populus trichocarpa*) by population transcriptome resequencing. Mol Ecol Resour.

[9] Tuskan GA, Difazio S, Jansson S, Bohlmann J, Grigoriev I, Hellsten U (2006). The genome of black cottonwood, *Populus trichocarpa* (Torr. & gray). Science.

[10] Paolucci I, Gaudet M, Jorge V, Beritognolo I, Terzoli S, Kuzminsky E (2010). Genetic linkage maps of *Populus alba* L. and comparative mapping analysis of sex determination across *Populus* species. Tree Genet Genomes.

[11] Yin T, DiFazio SP, Gunter LE, Riemenschneider D, Tuskan GA (2004). Large-scale heterospecific segregation distortion in *Populus* revealed by a dense genetic map. Theor Appl Genet.

[12] Gaudet M, Jorge V, Paolucci I, Beritognolo I, Mugnozza GS, Sabatti M (2008). Genetic linkage maps of *Populus nigra* L. including AFLPs, SSRs, SNPs, and sex trait. Tree Genet Genomes.

[13] Cervera MT, Storme V, Ivens B (2001). Dense genetic linkage maps of three *Populus* species (*Populus deltoids*, *P. nigra* and *P.trichocarpa*) based on AFLP and microsatellite markers. Genetics.

[14] Wang Y, Sun X, Tan B, Zhang B, Xu L, Huang M (2010). A genetic linkage map of *Populus adenopoda* maxim × *P. alba* L. hybrid based on SSR and SRAP markers. Euphytica.

[15] Woolbright SA, DiFazio S, Yin T, Martinsen GD, Zhang X, Allan GJ (2008). A dense linkage map of hybrid cottonwood (*Populus fremontii* × *P. angustifolia*) contributes to long-term ecological research and comparison mapping in a model forest tree. Heredity.

[16] Zhang B, Tong CF, Yin T, Zhang X, Zhuge Q, Huang M (2009). Detection of quantitative trait loci influencing growth trajectories of adventitious roots in *Populus* using functional mapping. Tree Genet Genomes.

[17] Pakull B, Groppe K, Meyer M, Markussen T, Fladung M (2009). Genetic linkage mapping in aspen (*Populus tremula* L. and *Populus tremuloides* Michx.). Tree Genet Genomes.

[18] Zhang D, Zhang Z, Yang K, Li B (2004). Genetic mapping in (*Populus tomentosa* × *Populus bolleana*) and *P. tomentosa* Carr. Using AFLP markers. Theor Appl Genet.

[19] Bradshaw HD, Stettler RF (1994). Molecular genetics of growth and development in *Populus*. II. Segregation distortion due to genetic load. Theor Appl Genet.

[20] Kuang H, Richardson T, Carson S, Wilcox P, Bongarten B (1999). Genetic analysis of inbreeding depression in plus tree 850.55 of *Pinus radiata* D. Don. I. Genetic map with distorted markers. Theor Appl Genet.

[21] Yang S, Yuan Y, Wang L, Li J, Wang W, Liu H (2012). Great majority of recombination events in *Arabidopsis* are gene conversion events. Proc Natl Acad Sci.

[22] Zickler D, Kleckner N (1999). Meiotic chromosomes: integrating structure and function. Annu Rev Genet.

[23] Lu PL, Han XW, Qi J, Yang JG, Wijeratne AJ, Li T (2012). Analysis of Arabidopsis genome-wide variations before and after meiosis and meiotic recombination by resequencing Landsberg erecta and all four products of a single meiosis. Genome Res.

[24] Chen JM, Cooper DN, Chuzhanova N, Ferec C, Patrinos GP (2007). Gene conversion: mechanisms, evolution and human disease. Nat Rev Genet.

[25] Ky CL, Barre P, Trouslot P, Akaffou S, Louarn J, Charrier A (2000). Interspecific genetic linkage map, segregation distortion and genetic conversion in coffee (Coffea sp.). Theor Appl Genet.

[26] Tong CF, Li HG, Wang Y, Li XR, Ou JJ, Wang DY (2016). Construction of high-density linkage maps of *Populus deltoides* × *P. simonii* using restriction-site associated DNA sequencing. PLoS One.

[27] Doyle JJ, Doyle JL (1987). A rapid DNA isolation procedure from small quantities of fresh leaf tissues. Phytolog Bull.

[28] Mousavi M, Tong CF, Liu FX, Tao ST, Wu JY, Li HG (2016). De novo SNP discovery and genetic linkage mapping in poplar using restriction site associated DNA and whole-genome sequencing technologies. BMC Genomics.

[29] Patel RK, Jain M (2012). NGS QC toolkit: a toolkit for quality control of next generation sequencing data. PLoS One.

[30] Li H, Durbin R (2009). Fast and accurate short read alignment with burrows-wheeler transform. Bioinformatics.

[31] Li H, Handsaker B, Wysoker A, Fennell T, Ruan J, Homer N (2009). The sequence alignment/map format and SAMtools. Bioinformatics.

[32] Kozarewa I, Ning Z, Quail MA, Sanders MJ, Berriman M, Turner DJ (2009). Amplification-free Illumina sequencing-library preparation facilitates improved mapping and assembly of (G+C)-biased genomes. Nat Methods.

[33] Ebbert MT, Wadsworth ME, Staley LA, Hoyt KL, Pickett B, Miller J (2016). Evaluating the necessity of PCR duplicate removal from next-generation sequencing data and a comparison of approaches. BMC Bioinformatics.

[34] Geraci F (2010). A comparison of several algorithms for the single individual SNP haplotyping reconstruction problem. Bioinformatics.

[35] Grattapaglia D, Sederoff R (1994). Genetic linkage maps of Eucalyptus grandis and Eucalyptus urophylla using a pseudo-testcross: mapping strategy and RAPD markers. Genetics.

[36] Judd SR, Petes TD (1988). Physical lengths of meiotic and mitotic gene conversion tracts in *Saccharomyces cerevisiae*. Genetics.

[37] Scheet P, Stephens M (2006). A fast and flexible statistical model for large-scale population genotype data: applications to inferring missing genotypes and haplotypic phase. Am J Hum Genet.

[38] Browning SR, Browning BL (2007). Rapid and accurate haplotype phasing and missing-data inference for whole-genome association studies by use of localized haplotype clustering. Am J Hum Genet.

[39] Delaneau O, Zagury JF, Marchini J (2013). Improved whole-chromosome phasing for disease and population genetic studies. Nat Methods.

[40] Loh PR, Danecek P, Palamara PF, Fuchsberger C, Reshef YA, Finucane HK (2016). Reference-based phasing using the haplotype reference consortium panel. Nat Genet.

[41] Liu EY, Morgan AP, Chesler EJ, Wang W, Churchill GA (2014). Pardo-Manuel de Villena F. High-resolution sex-specific linkage maps of the mouse reveal polarized distribution of crossovers in male germline. Genetics.

[42] Schadt EE, Turner S, Kasarskis A (2010). A window into third-generation sequencing. Hum Mol Genet.

