# Supplementary material for: Identification of recombination events in outbred species with next-generation sequencing data
Source: BMC Genomics. 2018 May 25;19:398. doi: 10.1186/s12864-018-4791-x (PMC5970487; doi:10.1186/s12864-018-4791-x)
Supplement: Supplementary file 1 — Table S1. Summary of parental blocks from the intermediate files of ‘parent1.abxaa.5snps.blocks’, ‘parent2.aaxab.5snps.blocks’, ‘parent1.long.haplotype’ and ‘parent2.long.haplotype’, created by findGCO. Table S2. The number of SNPs contained in the long parental haplotypes from the intermediate files of ‘parent1.long. Haplotype’ and ‘parent2.long.haplotype’ created by findGCO. Table S3. Distribution of the number of gene conversion events detected in each of the 10 progeny and inherited from the male parent based on the reference genome sequences. Figure S1. CO patterns identified in each progeny on all chromosomes in the female parent P. deltoides. Figure S2. CO patterns identified in each progeny on all chromosomes in the male parent P. simonii. (DOCX 277 kb) [file 12864_2018_4791_MOESM1_ESM.docx]

**Table S1** Summary of parental blocks from the intermediate files of ‘parent1.abxaa.5snps.blocks’, ‘parent2.aaxab.5snps.blocks’, ‘parent1.long.haplotype’ and ‘parent2.long.haplotype’, created by *findGCO*.

| Parent | *P. deltoides* (Female) | *P. simonii* (Male) |
| --- | --- | --- |
| Block | 54,753 | 35,458 |
| SNP | 647,973 | 427,863 |
| Average Length | 842 | 806 |
| Std | 912 | 935 |
| Min | 5 | 8 |
| Max | 26,133 | 20,230 |
| Coverage | 10.62% | 6.58% |

**Table S2** The number of SNPs contained in the long parental haplotypes from the intermediate files of ‘parent1.long. haplotype’ and ‘parent2.long.haplotype’ created by *findGCO*.

| Parent | *P. deltoides* (Female) | *P. simonii* (Male) |
| --- | --- | --- |
| SNP | 9,942 | 8,149 |
| SNP On Linkage Map | 1,205 | 700 |

**Table S3** Distribution of the number of gene conversion events detected in each of the 10 progeny and inherited from the male parent based on the reference genome sequences

| Ref. | Progeny ID | | | | | | | | | | Aver. |
| --- | --- | --- | --- | --- | --- | --- | --- | --- | --- | --- | --- |
|  | B35-2 | C25-3 | C3-2 | C32-2 | C5-3 | 3_12 | 3_14 | 3_15 | 3_16 | 3_18 |  |
| Chr01 | 530 | 507 | 580 | 548 | 584 | 557 | 535 | 573 | 553 | 587 | 555.4 |
| Chr02 | 156 | 171 | 156 | 162 | 182 | 153 | 174 | 192 | 173 | 163 | 168.2 |
| Chr03 | 219 | 225 | 196 | 206 | 205 | 206 | 198 | 177 | 246 | 223 | 210.1 |
| Chr04 | 188 | 195 | 178 | 201 | 216 | 209 | 195 | 214 | 166 | 195 | 195.7 |
| Chr05 | 150 | 166 | 167 | 174 | 185 | 180 | 158 | 163 | 158 | 179 | 168.0 |
| Chr06 | 170 | 184 | 197 | 190 | 194 | 192 | 171 | 187 | 177 | 208 | 187.0 |
| Chr07 | 115 | 109 | 121 | 110 | 118 | 116 | 119 | 119 | 115 | 129 | 117.1 |
| Chr08 | 144 | 126 | 132 | 141 | 142 | 149 | 136 | 136 | 163 | 125 | 139.4 |
| Chr09 | 75 | 99 | 81 | 90 | 82 | 92 | 94 | 94 | 92 | 107 | 90.6 |
| Chr10 | 124 | 139 | 157 | 140 | 162 | 145 | 138 | 121 | 127 | 136 | 138.9 |
| Chr11 | 148 | 146 | 165 | 183 | 159 | 157 | 165 | 167 | 158 | 179 | 162.7 |
| Chr12 | 91 | 100 | 105 | 93 | 113 | 104 | 111 | 121 | 103 | 106 | 104.7 |
| Chr13 | 134 | 129 | 152 | 156 | 145 | 152 | 165 | 136 | 139 | 156 | 146.4 |
| Chr14 | 194 | 191 | 202 | 201 | 201 | 190 | 184 | 203 | 206 | 210 | 198.2 |
| Chr15 | 136 | 139 | 135 | 134 | 139 | 133 | 134 | 124 | 135 | 150 | 135.9 |
| Chr16 | 145 | 129 | 140 | 138 | 127 | 122 | 132 | 129 | 125 | 139 | 132.6 |
| Chr17 | 182 | 187 | 191 | 175 | 202 | 194 | 203 | 207 | 174 | 188 | 190.3 |
| Chr18 | 164 | 123 | 158 | 151 | 134 | 155 | 126 | 156 | 134 | 149 | 145.0 |
| Chr19 | 161 | 177 | 182 | 183 | 189 | 193 | 191 | 175 | 172 | 160 | 178.3 |
| Scaff. | 189 | 216 | 192 | 194 | 200 | 206 | 186 | 200 | 190 | 222 | 199.5 |
| Total | 3415 | 3458 | 3587 | 3570 | 3679 | 3605 | 3515 | 3594 | 3506 | 3711 | 3564.0 |


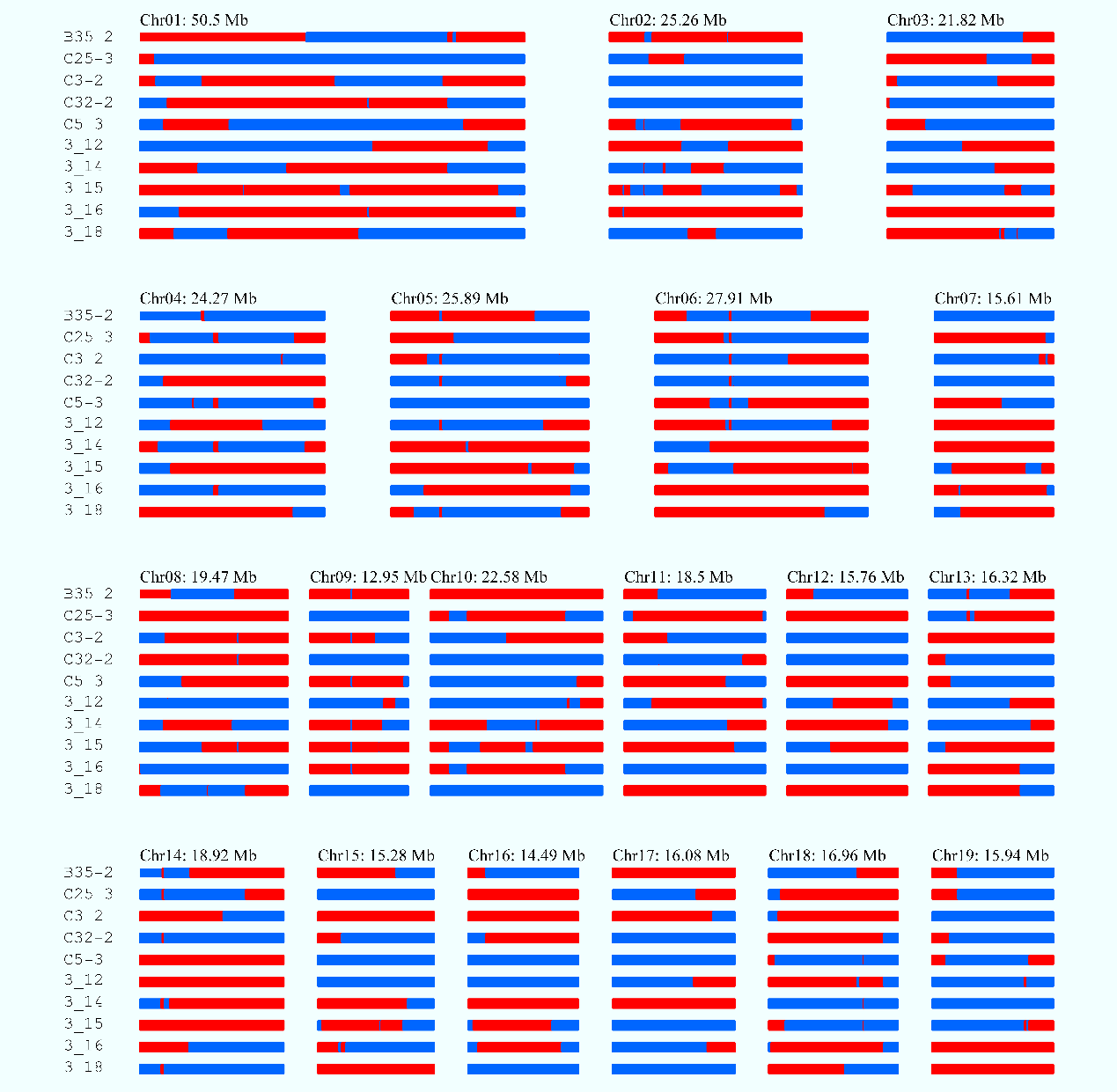


**Fig. S1** CO patterns identified in each progeny on all chromosomes in the female parent *P. deltoides*.


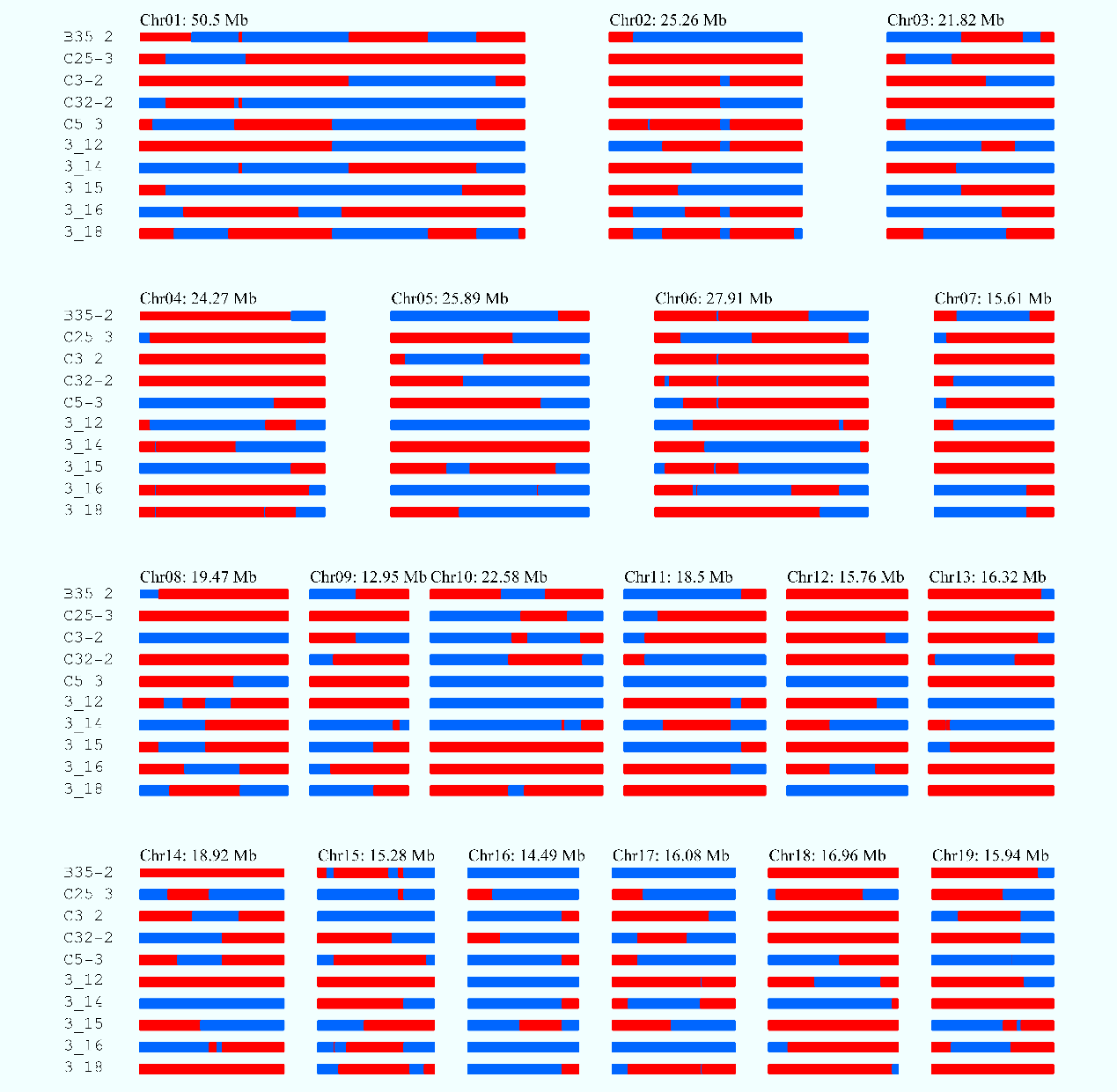


**Fig. S2** CO patterns identified in each progeny on all chromosomes in the male parent *P. simonii*.
